# Lactate to Albumin Ratio for Predicting Clinical Outcomes after In-Hospital Cardiac Arrest

**DOI:** 10.3390/jcm12124136

**Published:** 2023-06-19

**Authors:** Jafer Haschemi, Charlotte Theresia Müller, Jean Marc Haurand, Daniel Oehler, Maximilian Spieker, Amin Polzin, Malte Kelm, Patrick Horn

**Affiliations:** 1 Department of Cardiology, Pulmonology and Vascular Medicine, University Hospital Düsseldorf, Medical Faculty of the Heinrich Heine University Düsseldorf, 40225 Düsseldorf, Germany; jafer.haschemi@med.uni-duesseldorf.de (J.H.); charlotte.theresia.mueller@hhu.de (C.T.M.); jeanmarcstefan.haurand@med.uni-duesseldorf.de (J.M.H.); daniel.oehler@med.uni-duesseldorf.de (D.O.); maximilian.spieker@med.uni-duesseldorf.de (M.S.); amin.polzin@med.uni-duesseldorf.de (A.P.); malte.kelm@med.uni-duesseldorf.de (M.K.); 2 CARID, Cardiovascular Research Institute, Medical Faculty, University Hospital Düsseldorf, Heinrich-Heine University, 40225 Düsseldorf, Germany

**Keywords:** IHCA, resuscitation, cardiac life support, survival, marker

## Abstract

In-hospital cardiac arrest (IHCA) is associated with high mortality and poor neurological outcomes. Our objective was to assess whether the lactate-to-albumin ratio (LAR) can predict the outcomes in patients after IHCA. We retrospectively screened 75,987 hospitalised patients at a university hospital between 2015 and 2019. The primary endpoint was survival at 30-days. Neurological outcomes were assessed at 30 days using the cerebral performance category scale. 244 patients with IHCA and return of spontaneous circulation (ROSC) were included in this study and divided into quartiles of LAR. Overall, there were no differences in key baseline characteristics or rates of pre-existing comorbidities among the LAR quartiles. Patients with higher LAR had poorer survival after IHCA compared to patients with lower LAR: Q1, 70.4% of the patients; Q2, 50.8% of the patients; Q3, 26.2% of the patients; Q4, 6.6% of the patients (*p* = 0.001). Across increasing quartiles, the probability of a favourable neurological outcome in patients with ROSC after IHCA decreased: Q1: 49.2% of the patients; Q2: 32.8% of the patients; Q3: 14.7% of the patients; Q4: 3.2% of the patients (*p* = 0.001). The AUCs for predicting 30-days survival using the LAR were higher as compared to using a single measurement of lactate or albumin. The prognostic performance of LAR was superior to that of a single measurement of lactate or albumin for predicting survival after IHCA.

## 1. Introduction

In-hospital cardiac arrest (IHCA) is a major adverse event associated with high mortality and poor neurological outcomes [1,2,3]. Overall, the incidence is high with 1.5–2.8 cases per 1000 admissions in Europe [4]. A significant percentage of patients do not survive resuscitation attempts and fail to achieve stable spontaneous circulation. Moreover, there is also a notable proportion of patients who die during the post-resuscitation phase [5,6]. Only about 15–20% of patients with IHCA survive the event to discharge [6,7]. The post-resuscitation phase is characterised by intricate pathophysiological changes that arise from preceding ischemia, reperfusion, and the underlying cause of cardiac arrest. These complex alterations have the potential to trigger multiorgan failure [8].

Accurate prediction of outcomes after IHCA could provide critical information for physicians and relatives and help with appropriate treatment decisions. The timely anticipation of neurological outcomes holds significance in guiding subsequent therapeutic approaches. Deciding whether to continue, restrict, or terminate intensive care therapy presents a significant issue with substantial ethical and socioeconomic implications in routine clinical practice. To assess the prognosis following cardiac arrest, a multimodal approach involving clinical examination, neurophysiological evaluations, biomarkers, and imaging techniques should be employed [6,9]. 

Established biomarkers such as neuron-specific enolase (NSE), S100 protein B (S-100B), and the cytokine interleukin-8 (IL-8) are used for prognostication after cardiac arrest [10,11]. NSE is an enzyme primarily found in neurons of the brain and peripheral nervous system. Elevated levels of NSE in the bloodstream indicate cerebral damage [10]. When combined with clinical presentation, electrophysiology, and imaging techniques, NSE can play a significant role in assessing neurological outcomes [7]. The S-100B is a calcium-binding peptide primarily present in the nervous system, making it a potential additional marker for brain damage [11]. Cytokines, including IL-8, play a crucial role in the development of post-cardiac arrest syndrome. After cardiac arrest, the excessive release of circulating cytokines which is released by activated macrophages and endothelial cells contributes to ischemia/reperfusion injury, brain dysfunction, and myocardial dysfunction. Elevated levels of inflammatory cytokines have been linked to increased mortality rates and/or unfavorable neurological outcomes [10].

Moreover, increased lactate value, which may indicate hypoxia potentially in conjunction with the duration of no blood flow following cardiac arrest, is associated with increased mortality [12]. Albumin, produced in the liver, is the predominant protein found in blood plasma and plays a crucial role in various bodily functions. It contributes to the maintenance of oncotic pressure, possesses anti-inflammatory and antioxidant properties, and serves as an important transport protein. However, factors such as inflammation, critical illness, or liver disease can diminish its synthesis [13,14]. Consequently, studies have demonstrated that a decrease in serum albumin levels is linked to higher mortality rates following cardiac arrest [15].

The exploration of the ratio of inverse changes arising from distinct mechanisms in two independent predictors, specifically lactate and albumin, holds the potential for significant benefits in prognosticating the outcomes of patients following cardiac arrest. It was previously demonstrated that the lactate-albumin ratio (LAR) had a better predictive performance than lactate alone in terms of favourable neurological outcomes after OHCA [16]. However, it is well known the characteristics of patients with IHCA differ substantially from those of patients with OHCA [17,18]. Therefore, it is necessary to identify specific prognostic tools for IHCA. Our objective was to assess whether the LAR predicts outcomes in patients with a return of spontaneous circulation (ROSC) after IHCA.

## 2. Materials and Methods

### 2.1. Study Design

In this study, all data were analyzed retrospectively. At the time of submission to the ethics committee, all data had already been collected and were fully available in the hospital information system of the University Hospital Düsseldorf or the respective patient records.

This study was conducted in accordance with the guidelines of the Declaration of Helsinki and was approved by the University of Düsseldorf Committee on Human Research (Study number 2022-2273). The requirement for informed patient consent was waived by the University of Düsseldorf Committee on Human Research due to the use of anonymised data collected during critical care. Whenever it was feasible to reach out to them, every individual who survived provided written informed consent for the utilisation of their anonymous medical data relating to the defined hospitalisation.

### 2.2. Population

**Figure 1:**
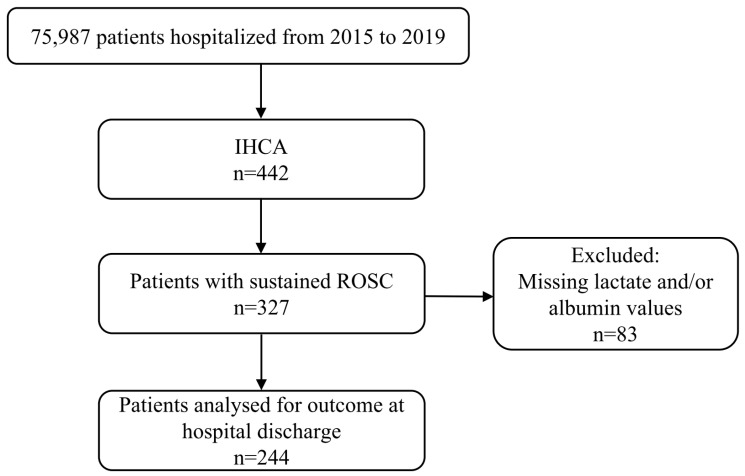
Flow chart of the study design. IHCA = Intra-hospital cardiac arrest, LAR = Lactate to albumin ratio, Q = Quartile.

We retrospectively screened 75,987 patients who had been hospitalised in the internal medicine or neurological general ward from 2015 to 2019 at the University Hospital of Dusseldorf for IHCA occurrence. Patients who achieved ROSC and had lactate and albumin levels measured within one hour after ROSC were included in this study. After ROSC, the evaluation of lactate and albumin was standardised. Blood sampling was usually performed immediately after reaching ROSC in the intensive care unit, but the subsequent assessments of these variables during the clinical course were conducted at different time points. To eliminate the possibility of sampling time bias, we only included data from the standardised time point in our analysis. Patients who did not have a lactate and/or albumin value measured within the first hour were excluded from the study (Figure 1).

Patient characteristics, baseline data, and data related to the events were assessed using patients’ charts and medical records.

LAR was calculated as the ratio of lactate (measured in mmol/L) to albumin (measured in g/dL). Depending on the LAR value, patients were divided into 4 quartiles. The first quartile, Q1, included patients with the lowest LAR, and the patients in the fourth quartile, Q4, had the patients with the highest LAR. Accordingly, the study population was divided into four groups (Q1–Q4).

The primary endpoint of this study was 30-day survival. The secondary endpoint was favourable neurological outcomes at 30 days.

### 2.3. Cerebral Performance Category

Neurological outcomes were assessed using the cerebral performance category (CPC) scale, a five-point scale that assesses brain recovery by evaluating functional and cognitive aspects of brain function [19]. The CPC scale was determined by a chart review of the neurological assessment performed by a physician at the time of discharge. In cases of missing chart reviews, a structured interview was conducted. A favourable neurological outcome was defined as a CPC of 1 or 2 at 30 days, and CPC 3 to 5 are considered poor neurologic outcomes or brain death. [19].

### 2.4. Statistical Analysis

Statistical analyses were performed using SPSS statistics, version 28 (IBM, Armonk, NY, USA), MedCalc (Version 20.11, Ostend, Belgium), and GraphPad Prism^®^ version 7.0 (Graph Pad Software, San Diego, CA, USA).

Categorical variables were reported as absolute values and percentages. Continuous data were expressed as median (interquartile range [IQR]). The interquartile range was calculated as the measure of dispersion.

Categorical data were compared using the χ2 test. The D’Agostino & Pearson omnibus normality test was used to assess the normal distribution of continuous variables. Student’s unpaired *t*-test (in the case of a normal distribution) or Mann-Whitney U test (in the case that continuous variables did not follow a normal distribution) were performed to compare the means between the two groups. The 30-day survival was analyzed using the Kaplan-Meier method. The survival rates of all four groups were then compared using logrank tests.

Logistic regression analysis was used to identify predictors of survival to discharge and favourable neurological outcomes after IHCA. Variables with a *p*-value < 0.10, in the univariate analysis and variables known or assumed to be associated with survival after IHCA were included in the multivariable model. Receiver operating characteristic (ROC) curve analysis was performed to determine the area under the curve (AUC) of the variables for predicting survival. The Delong test was performed to compare the AUC of different variables. The Youden’s Index was used to balance the sensitivity and specificity of the prediction of 30-day survival and favourable neurological outcomes. Statistical significance was set at *p* < 0.05.

## 3. Results

### 3.1. Patients’ Characteristics

From 2015 to 2019, 75,987 patients were hospitalised in the internal medicine or neurology departments of the University Hospital Dusseldorf, Germany. During this period, 442 patients experienced IHCA. A total of 327 (74%) of the IHCA patients achieved ROSC. Of these patients, 83 were excluded from the study due to incomplete data sets.

A total of 244 patients were included in this study, divided into quartiles of LAR (Q1: 0.54 [IQR 0.38–0.70]; Q2: 1.64 [IQR 1.22–2.12]; Q3: 3.39 [IQR 2.71–3.78]; Q4: 5.93 [IQR 5.16–8.19]).

According to the division into four quartiles, the lactate values were higher from quartile to quartile (Q1: 1.7 [IQR 1.2–2.4] mmol/L; Q2: 5.0 [IQR 4.2–6.5] mmol/L; Q3: 9.3 [IQR 7.7–10.9] mmol/L; Q4: 14.0 [IQR 12.3–19.3] mmol/L, *p* < 0.001). Conversely, albumin values were lower from quartile to quartile. (Q1: 3.7 [IQR 3.0–3.9] g/dL; Q2: 3.3 [IQR 2.9–3.7] g/dL; Q3: 2.8 [IQR 2.5–3.5] g/dL; Q4: 2.3 [IQR 1.8–2.8] g/dL, *p* < 0.001).

Overall, there were no differences in key baseline characteristics, rates of preexisting comorbidities, and Charlson comorbidity index among the LAR quartiles (Table 1).

The median age was 73 (IQR 65–81) years in the Q1 group, 75 (IQR 64–81) years in the Q2 group, 71 (IQR 64–80) years in the Q3 group, and 71 (IQR 60–81) years in the Q4 group (*p* = 0.856). There were predominantly male patients in all four groups (62% in the Q1 group, 67% in the Q2 group, 64% in the Q3 group, and 52%, in the Q4 group, *p* = 0.374).

Most patients suffered from preexisting cardiovascular disease with no differences in all groups. CAD was present in 61% of the Q1 group, 77% of the Q2 group, 61% of the Q3 group, and 54% of the Q4 group (*p* = 0.057) Most patients suffered from arterial hypertension: Q1: 75% of the patients; Q2: 84% of the patients; Q3: 75% of the patients; Q4: 77% of the patients, *p* = 0.656. There was no difference in the prevalence of liver cirrhosis in all four groups: Q1: 2% of the patients; Q2: 7% of the patients; Q3: 5% of the patients; Q4: 2% of the patients, *p* = 0.374.

Haemoglobin levels decreased with increasing LAR quartiles (Q1:12.2 [IQR 9.8–13.9] g/dL; Q2: [11.4 (IQR 9.9–13.9] g/dL; Q3: [10.4 (IQR 8.7–12.6] g/dL; Q4: 9.7 [IQR 7.9–11.1] g/dL, *p* = 0.001) while the C-reactive protein level increased as the LAR quartile increased (Q1: 1.8 [0.6–5.4] mg/dL; Q2: 2.2 [0.6–8.9] mg/dL; Q3: 4.1 [1.2, 9.3] mg/dL; Q4: 5.1 [1.4, 11.6] mg/dL, *p* = 0.005).

The admission diagnosis, aetiology, and time point of cardiac arrest were similar among the groups (Table 1). The rate of shockable primary rhythm was lower in patients in the higher quartile: Q1: 52% of the patients; Q2: 46% of the patients; Q3: 31% of the patients; Q4: 29% of the patients, *p* = 0.007.

The time to achieve ROSC was longer with increasing LAR quartiles: 4 (IQR 1–10) min in Q1. 10 (IQR 3–20) min in Q2, 15 (IQR 6–30) min in Q3, and 25 (IQR 20–30) min in Q4, *p* < 0.001).

D-dimer, troponin T, and peak NSE levels were higher in the higher LAR quartiles (Table 2).

### 3.2. The Outcome of Patients after IHCA

**Figure 2:**
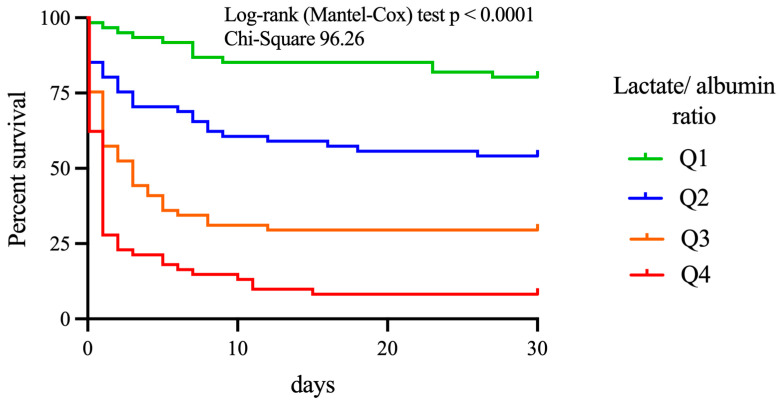
The 30-day survival after IHCA. Kaplan-Meier curve for the survival of the four groups: Q1–Q4. 30-day survival was lower with higher LAR. IHCA = In-hospital cardiac arrest; Q = quartile; LAR = Lactate to albumin ratio.

In the full cohort, 94 out of 244 patients (38.5%) survived for at least 30 days after IHCA. Patients with a higher LAR had poorer survival after IHCA than those with a lower LAR. The probability of 30-day survival in patients with ROSC after IHCA decreased in a stepwise manner across increasing quartiles: Q1, 43 out of 61 patients (70.4%); Q2, 31 out of 61 patients (50.8%); Q3, 16 out of 61 patients (26.2%); Q4, 4 out of 61 patients (6.6%), (*p* = 0.001 among the groups). The Kaplan-Meier curve and log-rank (Mantel-Cox) test confirmed lower survival of patients with higher LAR (Figure 2).

**Figure 3:**
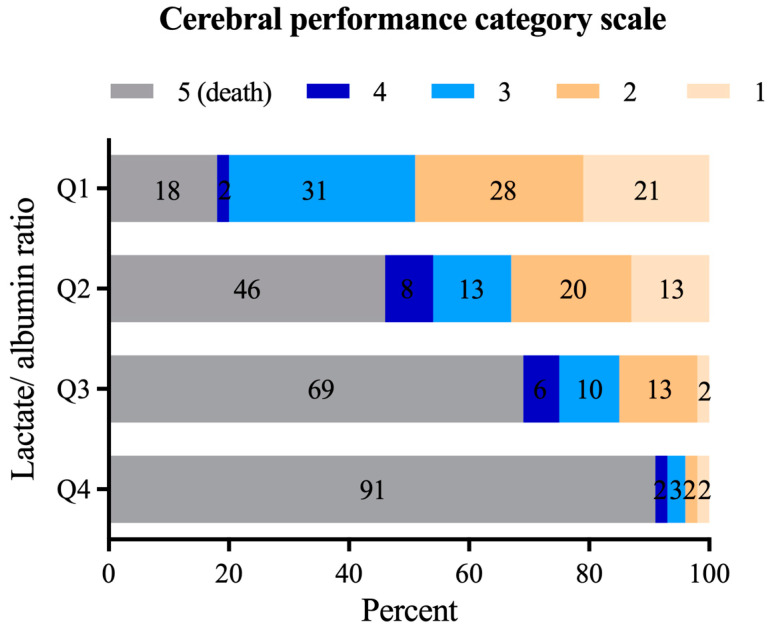
Neurological outcome according to the CPC scale after IHCA.

In the full cohort, 63 out of 244 patients (25.8%) survived IHCA with a favourable neurological outcome. Patients with a higher LAR had poorer neurological outcomes after IHCA than those with a lower LAR (Figure 3). Across increasing quartiles, the probability of a favourable neurological outcome (CPC 1 + 2) in patients with ROSC after IHCA decreased: Q1, 30 out of 61 patients (49.2%); Q2, 20 out of 61 patients (32.8%); Q3, 9 out of 61 patients (14.7%); Q4, 4 out of 61 (3.2%), (*p* = 0.001 among the groups, *p* < 0.001 between Q1 vs Q3 or Q4; *p* < 0.001 between Q2 vs. Q4).

A CPC scale of 1 or 2 indicates a good neurological outcome. Patients with a higher LAR had worse neurological outcomes than those with a lower LAR. IHCA = In-hospital cardiac arrest; CPC = Cerebral Performance Category; LAR = Lactate to albumin ratio

We performed a regression analysis using the full cohort to identify the predictors of 30-day survival after IHCA. In multivariate analysis, the presence of a shockable primary rhythm, higher haemoglobin level, and lower LAR (Odds ratio [OR] 0.616, 95% confidence interval [CI] 0.422–0.899, *p* = 0.012) were independent predictors of survival (Table A1). The OR for lactate was 0.822 (95% CI 0.705–0.959, *p* = 0.013) when it was included instead of LAR. For albumin, the OR was 1.837 (95% CI 0.926–3.643, *p* = 0.082).

In addition, multivariate analysis demonstrated that a low LAR (OR 0.734, CI 0.540–0.997, *p* = 0.048) was an independent predictor of favourable neurological outcomes (Table A2). In the analysis of multivariate regression for predicting favorable neurological outcomes, the OR for lactate was 0.871 (95% CI 0.765–0.992, *p* = 0.037) when it was included instead of LAR, while the OR for albumin was 1.262 (95% CI 0.613–2.598, *p* = 0.527).

**Figure 4:**
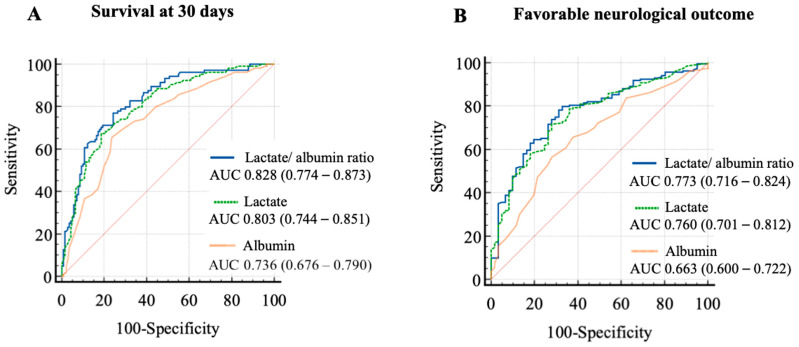
ROC curve analysis of LAR, Lactate, and Albumin for predicting 30-day survival and neurological outcome after IHCA. (**A**) The AUC for predicting 30-day survival using the LAR was higher as compared to using a single measurement of lactate or albumin. (**B**) The AUC for predicting favourable neurologic outcomes using the LAR was higher as compared to using a single measurement of lactate or albumin. ROC = Receiver operating characteristics; IHCA = In-hospital cardiac arrest; AUC = Area under the curve; LAR = Lactate to albumin ratio.

The AUCs for predicting 30-day survival using the LAR were higher (0.828 [CI 0.774–0.873]), as compared to using a single measurement of lactate (0.803 [CI 0.747–0.851], *p* = 0.011), or a single measurement of albumin (0.736 [CI 0.676–0.790], *p* = 0.004) (Figure 4A). The AUC for predicting 30-day survival using NSE peak level was AUC 0.738 (CI 0.662–0.804).

Similarly, the AUC for predicting a favourable neurologic outcome using LAR was 0.773 (CI 0.716–0.824), as compared to using a single measurement of lactate (0.760 [CI 0.701–0.812], *p* = 0.189), or albumin (0.663 [CI 0.600–0.722], *p* = 0.004) (Figure 4B). The AUC for predicting a favorable neurologic outcome using the NSE peak level was 0.706 (CI 0.629–0.776).

Using a cut-off value of 2.3 for LAR, the sensitivity to predict 30-day survival after IHCA was 77% (68–85%), with a specificity of 76% (68–83%) (Youden’s Index, 0.526).

The optimal value for a single measurement of albumin to predict 30-day survival after IHCA was 3.1 g/dL (sensitivity 65 [55–74%] %, specificity 76% [69–83%] (Youden’s Index, 0.418).

The optimal value for a single measurement of lactate was 5 mg/L (sensitivity 67% (57–76%), specificity 81% (74–88%) (Youden’s Index, 0.487).

Using a cut-off value of 1.26 LAR, the sensitivity for predicting favourable neurological outcomes after IHCA was 80% (73–85%), with a specificity of 67% (5479%) (Youden’s Index, 0.470).

The cut-off value for a single measurement of albumin to predict favourable neurological outcome was 2.9 g/dL (sensitivity 56% [49–64%], specificity 72% [60–83%], Youden’s Index 0.282).

The cut-off value for a single measurement of lactate to predict favourable neurological outcomes were 5 mg/L (sensitivity 72% [65–78%], specificity 72% [60–83%], and Youden’s Index 0.432).

## 4. Discussion

This study demonstrated that a low LAR immediately after ROSC was associated with increased survival and favourable neurological outcomes. The prognostic performance of the LAR might be superior to that of a single measurement of lactate or albumin for predicting survival or favourable neurologic outcomes after IHCA.

Outcomes after IHCA are affected by patient characteristics [3,20,21,22], the time point of cardiac arrest, and the time to ROSC [3,21,23,24]. It has been previously demonstrated that patients who are witnessed or monitored at the time of a cardiac arrest have a significantly higher survival rate to hospital discharge than those who are neither monitored nor witnessed [24,25,26].

In the present study, the presence of a shockable primary rhythm, higher haemoglobin level, and lower LAR were independent predictors of 30d survival. The presence of a non-shockable rhythm may reflect the delayed detection of initial shockable rhythms that progress to non-shockable rhythms. There is some evidence obtained from other studies to suggest that lower haemoglobin levels following cardiac arrest are associated with poor outcomes [27,28]. Tissue oxygen delivery is dependent on hemoglobin level, and cerebral oxygenation can be compromised by anaemia, particularly following an acute brain injury [29]. Low hemoglobin levels could exacerbate the effects of oxygen deprivation during cardiac arrest and reperfusion injury. It is already known that hyperoxia after ROSC is associated with increased mortality in both OHCA and IHCA [30,31]. However, in our study, no variation in arterial oxygen partial pressure was observed across all four groups, allowing us to exclude this factor as a potential cause for the observed outcomes. In addition, utilizing capillary refill time (CRT) as a means to evaluate the perfusion status of critically ill patients at the bedside could serve as a valuable tool in guiding resuscitation efforts [32,33]. The impact of CRT on predicting the outcome following cardiac arrest remains uncertain. Unfortunately, our study lacks information regarding the CRT of the patients, which prevents us from making any assertions about a potential association between LAR and CRT.

Accurate prediction of survival and favourable neurological outcomes after IHCA could provide critical information for physicians and relatives and could help with appropriate treatment decisions. Prediction tools such as the Good Outcome Following Attempted Resuscitation (GO FAR 2) score provide clinicians with a prognostic estimate of the likelihood of a good neurological outcome after IHCA based on pre-arrest patient factors [34,35]. However, calculating the GO-FAR score requires more than ten variables, and it has shown the AUC to predict good or moderate neurological outcomes after an OHCA of 0.7 [34,36].

In contrast, the LAR is easy to determine because only two parameters are required. Kong et al. analysed the prognostic value of LAR in patients after out-of-hospital cardiac arrest. They demonstrated that LAR had a better predictive performance than lactate alone in terms of favourable neurological outcomes and survival after OHCA [16]. Here, we demonstrated, for the first time, the prognostic value of LAR for predicting clinical outcomes in patients with in-hospital cardiac arrest. In the present study, the AUC for predicting survival after IHCA was 0.83, and 0.77 for predicting good neurological outcomes. Thus, LAR was superior in predicting survival and favourable neurological outcomes after IHCA than using a single measurement of lactate or albumin.

Increased lactate levels are associated with mortality and are widely used for early diagnosis, management, and risk stratification in critically ill patients [37]. High levels of lactate are also associated with lower survival after IHCA [12,38] and, after a cardiac arrest, are related to tissue hypoperfusion and ischaemia-reperfusion [38]. However, lactate levels can be affected by several different conditions, including decreased lactate elimination due to hepatic or renal dysfunction as well as accelerated glycolysis, and the diagnostic value of the initial lactate level alone might be low [39,40,41].

Low serum albumin is significantly associated with mortality and morbidity in critically ill patients and after IHCA [15,42,43]. Human serum albumin possesses protective effects through anti-inflammatory properties and reduction of ischaemia-reperfusion injury, both of which might be important to counterbalance the post-resuscitation inflammatory response [44,45,46,47]. Similar to lactate, serum albumin levels are affected by multiple conditions, including inflammation, malnutrition, and liver cirrhosis [48]. The decreased level of albumin may be due to albumin loss in the urine in renal diseases/protein-losing gastropathy, decreased hepatic albumin synthesis, or protein malnutrition dysfunction [49,50,51].

The comprehensive combination of lactate and albumin analysis can provide potential benefits for predicting the prognosis of patients with ROSC after cardiac arrest using the ratio of inverse change due to the different mechanisms of the two independent predictors [52]. The combination of lactate as an expression for hypoxia and possibly the no-flow time after cardiac arrest and albumin as an expression for the status of the critically ill patient seems to have a predictive value for in-hospital mortality. Our study did not find any significant differences in the incidence of liver cirrhosis, renal function, or medical conditions assessed by the Charlson comorbidity score among the groups. Nevertheless, we believe that the patients varied in terms of their nutritional status and inflammation level, and the LAR measurement serves as an appropriate surrogate marker for these factors and can differentiate patients in terms of intrahospital survival and neurological outcome.

Routine monitoring of albumin and lactate levels after IHCA should be established in the clinical routine, as it can support risk stratification in post-resuscitation care.

There were limitations in this study. The present study was a single-centre study that included patients in an internal medicine or neurology non-intensive care unit ward; therefore, the interpretation of the results may not be generalised and transferred to other medical disciplines. In addition, the limited number of patients included in the study constrained the multivariate analysis.

In conclusion, the prognostic performance of LAR might be superior to that of a single measurement of lactate or albumin for predicting 30-day survival or favourable neurologic outcomes after IHCA.

## Figures and Tables

**Table 1:** Baseline characteristics of the study cohort. Categorical variables are reported as absolute values and percentages, whereas continuous data are expressed as medians with interquartile ranges. * indicates *p* < 0.012 compared to Q1; # indicates *p* < 0.012 compared to Q2.; ° indicates *p* < 0.012 compared to Q3.

Patient Characteristics	Complete Cohort (*n* = 244)	Q1 Group (*n* = 61)	Q2 Group (*n* = 61)	Q3 Group (*n* = 61)	Q4 Group (*n* = 61)	*p*-Value
LAR, median	2.47 (0.96, 4.17)	0.54 (0.38, 0.70)	1.64 (1.22, 2.12)	3.39 (2.71, 3.78)	5.93 (5.16, 8.19)	<0.001
Lactate after ROSC, median in mmol/L	7.0 (3.3, 11.3)	1.7 (1.2, 2.4)	5.0 (4.2, 6.5) *	9.3 (7.7, 10.9) *#	14.0 (12.3, 19.3) *#°	<0.001
Albumin, median in g/dL	3.0 (2.4, 3.6)	3.7 (3.0, 3.9)	3.3 (2.9, 3.7)	2.8 (2.5, 3.5) *#	2.3 (1.8, 2.8) *#°	<0.001
Age, median, years	73 (63, 80)	73 (65, 81)	75 (60, 81)	71 (64, 80)	71 (60, 81)	0.856
Women/male, *n*/*n* (%/%)	94/150 (39/61)	23/38 (38/62)	20/41 (33/67)	22/39 (36/64)	29/32 (48/52)	0.374
CAD, *n* (%)	150 (61)	37 (61)	47 (77)	37 (61)	33 (54)	0.057
PAD, *n* (%)	37 (15)	7 (11)	9 (15)	13 (21)	8 (13)	0.450
Arterial hypertension, *n* (%)	190 (78)	46 (75)	51 (84)	46 (75)	47 (77)	0.656
Diabetes mellitus, *n* (%)	66 (27)	16 (26)	16 (26)	16 (26)	18 (30)	0.969
Liver cirrhosis, *n* (%)	9 (4)	1 (2)	4 (7)	3 (5)	1 (2)	0.374
Hepatis B or C, *n* (%)	4 (2)	0 (0)	1 (2)	2 (3)	1 (2)	
GFR, median, mL/min	45 (27, 70)	44 (32, 74)	49 (23, 79)	47 (34, 77)	40 (24, 54)	0.205
Charlson Comorbidity Index	5 (4, 7)	5 (4, 6)	5 (4, 7)	6 (4, 8)	5 (4, 7)	0.236
Hemoglobin, median, g/dL	10.9 (9.1, 13.0)	12.2 (9.8, 13.9)	11.4 (9.9, 13.9)	10.4 (8.7, 12.6) *	9.7 (7.9, 11.1) *#	<0.001
C-reactive protein, median, mg/dL	3.2 (0.8, 8.9)	1.8 (0.6, 5.4)	2.2 (0.6, 8.9)	4.1 (1.2, 9.3) *	5.1 (1.4, 11.6) *	0.005
Elective admission, *n* (%)	31 (13)	11 (18)	6 (10)	11 (18)	3 (5)	0.075
Admission diagnosis						
Pneumonia, *n* (%)	25 (10)	5 (8)	3 (5)	13 (21)	4 (7)	
Acute heart failure, *n* (%)	21 (9)	4 (7)	5 (8)	5 (8)	7 (11)	
Acute coronary syndrome, *n* (%)	52 (21)	12 (20)	20 (33)	10 (16)	10 (16)	
Acute kidney failure, *n* (%)	14 (6)	5 (8)	2 (3)	2 (3)	5 (8)	
Cardiac arrhythmia, *n* (%)	19 (8)	3 (5)	3 (5)	4 (7)	9 (15)	
Gastrointestinal, *n* (%)	15 (6)	4 (7)	3 (5)	4 (7)	4 (7)	
Sepsis, *n* (%)	8 (3)	4 (7)	2 (3)	2 (3)	0 (0)	
Malignancy, *n* (%)	25 (10)	10 (16)	6 (10)	3 (5)	6 (10)	
Neurology, *n* (%)	21 (9)	3 (5)	3 (5)	8 (13)	7 (11)	
Peripheral artery disease, *n* (%)	2 (1)	1 (2)	1 (2)	0 (0)	0 (0)	
Pulmonary embolism, *n* (%)	3 (1)	1 (2)	2 (3)	0 (0)	0 (0)	
Other, *n* (%)	40 (16)	9 (15)	11 (18)	10 (16)	10 (16)	
Cardiac arrest characteristics						
Non-cardiac origin of IHCA, *n* (%)	122 (50)	26 (43)	26 (43)	34 (56)	36 (59)	0.142
Arrest time off-hours, *n* (%)	93 (38)	20 (33)	25 (41)	20 (33)	28 (46)	0.355

LAR = Lactate to albumin ratio; CAD = Coronary artery disease, PAD = Peripheral arterial disease, CKD = Chronic kidney disease, GFR = Glomerular fraction rate; Arrest time off-hours = Cardiac arrest that occurred during night shifts (>6 p.m. to <8 a.m.), weekends, and holidays, respectively.

**Table 2:** Intra-hospital outcome after IHCA. Categorical variables are reported as absolute values and percentages, whereas continuous data are expressed as medians with interquartile ranges. * indicates *p* < 0.012 compared to Q1; # indicates *p* < 0.012 compared to Q2; ° indicates *p* < 0.012 compared to Q3.

Patient Characteristics	Complete Cohort (*n* = 244)	Q1 Group (*n* = 61)	Q2 Group (*n* = 61)	Q3 Group (*n* = 61)	Q4 Group (*n* = 61)	*p*-Value
Primary shockable rhythm, *n* (%)	91 (37)	32 (52)	28 (46)	19 (31)	12 (29) *#	0.007
Defibrillation, *n* (%)	93 (38.1)	32 (52)	28 (46)	19 (31)	14 (30) *	0.003
Number of shocks performed in case of defibrillation, *n*	1 (1, 1)	(1, 1)	1 (1, 1)	1 (1, 2)	1 (1, 2)	0.257
Number of epinephrine applications, *n*	2 (1, 4)	0 (0, 1)	1 (0, 3) *	2 (1, 4) *	3 (2, 4) *#	0.001
Time to ROSC, median, min	11 (4, 25)	4 (1, 10)	10 (3, 20) *	15 (6, 30) *	25 (20, 30) *#°	0.001
Phosphate, median, mmol/L	1.7 (1.2, 2.5)	1.2 (0.9, 1.6)	1.5 (1.2, 2.2)	1.7 (1.1, 2.3) *	2.4 (1.8, 3.1) *#°	0.001
TroponinT, median, ng/L	135 (52, 431)	69 (37, 207)	100 (52, 672)	136 (65, 720) *	215 (108, 1020) *	0.001
NSE peak, median, µg/L	45 (30, 88)	37 (35, 142)	37 (21, 153)	39 (33, 293)	115 (63, 268) *#	0.003
D-Dimere, median, I/U	8.7 (3.1, 30.0)	4.0 (1.7, 9.0)	5.0 (2.6, 16.2)	11.0 (3.6, 30) *	20.0 (10.6, 30.0) *#	0.001
pH after ROSC, median	7.2 (7.1, 7.3)	7.4 (7.3, 7.4)	7.3 (7.2, 7.3) *	7.2 (7.0, 7.3) *#	7.1 (7.0, 7.2) *#	0.001
PaO_2_, median, mmHg	89 (77, 110)	88 (78, 104)	87 (77, 107)	91 (77, 111)	92 (78, 120)	0.657
Target temperature management, *n* (%)	240 (98.4%)	60 (98.4)	59 (96.7)	61 (100)	60 (98.4)	1
PCI after ROSC, *n* (%)	66 (27)	17 (68)	19 (31)	16 (26)	14 (23)	0.782
ECLS, *n* (%)	9 (4)	3 (5)	4 (7)	1 (2)	1 (2)	0.374

IHCA = in-hospital cardiac arrest; ROSC = return of spontaneous circulation; NSE = neuron-specific enolase; PCI = percutaneous coronary intervention; ECLS = extracorporeal circulatory life support.

## Data Availability

The data presented in this study are available on request from the corresponding author. The data are not publicly available due to ethical reasons.

## Appendix A

**Table A1:** Logistic regression analysis of the full cohort (patients with ROSC after IHCA) for 30-day survival, * indicates *p* < 0.05.

Patient Characteristics	Univariate			Multivariate		
	OR	95% CI	*p*-Value	OR	95% CI	*p*-Value
Age, years	0.971	0.949–0.992	0.008 *	0.976	0.935–1.009	0.267
Sex (female)	0.864	0.512–1.457	0.583			
Diabetes mellitus	0.908	0.512–1.611	0.742			
Hypertension	0.750	0.409–1.376	0.353			
CAD	2.158	1.249–3.727	0.006 *			
PAD	0.791	0.386–1.624	0.523			
Chronic kidney disease	0.739	0.572–0.954	0.020 *			
Charlson Comorbidity Index	0.897	0.804–1.001	0.053 *			
GFR, mL/min	1.018	1.008–1.028	0.001 *	1.007	0.987–1.027	0.497
Haemoglobin, g/dL	1.259	1.133–1.399	0.001 *	1.246	1.008–1.541	0.042 *
elective admission	1.759	0.824–3.754	0.145			
cardiac origin of IHCA	2.262	1.346–3.800	0.002 *	0.483	0.134–1.733	0.264
Arrest time on-hours, min	1.614	0.948–2.746	0.078	2.374	0.711–7.926	0.160
Primary shockable rhythm	4.425	2.545–7.695	0.001 *	3.906	1.039–14.689	0.044 *
Defibrillation, *n* (%)	4.049	2.341–7.002	0.001 *			
Number of epinephrine applications, *n*	0.569	0.472–0.686	0.001 *			
Time to ROSC	0.928	0.903–0.953	0.001 *	0.442	0.953–1.021	0.442
ECLS	1.717	0.450–6.558	0.429			
PCI after ROSC	2.550	1.430–4.549	0.002 *			
Lactate after ROSC	0.776	0.720–0.835	0.001 *			
Albumin after ROSC	2.213	1.535–3.190	0.001 *			
LAR after ROSC	0.498	0.407–0.610	0.001 *	0.616	0.422–0.899	0.012 *
Troponin after ROSC	1.000	1.000–1.000	0.113			
NSE peak level	0.983	0.974–0.993	0.001 *	0.991	0.980–1.012	0.119

IHCA = In-hospital cardiac arrest; OR = Odds ratio, CI = Confidence interval, GFR = Glomerular fraction rate, CAD = Coronary artery disease, PAD = peripheral arterial disease; ROSC = Return of spontaneous circulation; LAR = Lactate to albumin ratio; NSE = neuron-specific enolase; PCI = percutaneous coronary intervention; ECLS = extracorporeal circulatory life support; Arrest time on-hours = Cardiac arrest that occurred from Monday through Friday 8 a.m. to 6 p.m.

## Appendix B

**Table A2:** Logistic regression analysis of the full cohort (patients with ROSC after IHCA) for favorable neurological outcome * indicates *p* < 0.05.

Patient Characteristics	Univariate			Multivariate		
	OR	95% CI	*p*-Value	OR	95% CI	*p*-Value
Age, years	0.963	0.940–0.987	0.002 *	0.964	0.920–1.011	0.136
Sex (female)	0.882	0.483–1.609	0.682			
Diabetes mellitus	0.677	0.339–1.353	0.269			
Hypertension	0.660	0.339–1.286	0.222			
CAD	1.281	0.695–2.364	0.269			
PAD	0.657	0.273–1.582	0.349			
Chronic kidney disease	0.662	0.484–0.904	0.010 *			
Charlson Comorbidity Index	0.836	0.734–0.952	0.007 *	0.939	0.731–1.207	0.625
GFR, mL/min	1.017	1.006–1.029	0.002 *	0.998	0.977–1.020	0.857
Haemoglobin, g/dL	1.188	1.061–1.331	0.003 *	1.186	0.969–1.453	0.098
elective admission	1.503	0.665–3.400	0.328			
cardiac origin of IHCA	2.162	1.188–3.934	0.012 *	1.987	0.636–6.155	0.239
Arrest time on-hours	1.373	0.745–2.530	0.309	2.159	0.682–6.831	0.190
Primary shockable rhythm	1.603	0.889–2.888	0.116	0.698	0.218–2.230	0.544
Defibrillation, *n* (%)	1.514	0.841–2.726	0.167			
Number of epinephrine applications, *n*	0.631	0.512–0.776	0.001 *			
Time to ROSC	0.951	0.925–0.978	0.001 *	1.016	0.983–1.050	0.351
ECLS	0.839	0.171–4.193	0.429			
Lactate after ROSC	0.800	0.736–0.870	0.001 *			
Albumin after ROSC	2.133	1.412–3.224	0.001 *			
LAR after ROSC	0.577	0.444–0.698	0.001 *	0	0.540–0.997	0.048 *
Troponine after ROSC	1.000	1.000–1.000	0.304			
NSE peak level	0.989	0.979–0.999	0.034 *	0.992	0.981–1.003	0.170

IHCA = In-hospital cardiac arrest; OR = Odds ratio, CI = Confidence interval, GFR = Glomerular fraction rate, CAD = Coronary artery disease, PAD = peripheral arterial disease; ROSC = Return of spontaneous circulation; LAR = Lactate to albumin ratio; NSE = neuron-specific enolase; PCI = percutaneous coronary intervention; ECLS = extracorporeal circulatory life support; Arrest time on-hours = Cardiac arrest that occurred from Monday through Friday 8 a.m. to 6 p.m.
